# Functional Ecology of External Secretory Structures in *Rivea ornata* (Roxb.) Choisy (Convolvulaceae)

**DOI:** 10.3390/plants11152068

**Published:** 2022-08-08

**Authors:** Natthaphong Chitchak, Alyssa B. Stewart, Paweena Traiperm

**Affiliations:** Department of Plant Science, Faculty of Science, Mahidol University, Bangkok 10400, Thailand

**Keywords:** plant–animal interaction, micromorphology, anatomy, histochemistry, plant defense, pollination

## Abstract

Plants have evolved numerous secretory structures that fulfill diverse roles and shape their interactions with other organisms. *Rivea ornata* (Roxb.) Choisy (Convolvulaceae) is one species that possesses various external secretory organs hypothesized to be ecologically important. This study, therefore, aimed to investigate five secretory structures (nectary disc, petiolar nectaries, calycinal glands, staminal hairs, and foliar glands) using micromorphology, anatomy, histochemistry, and field observations of plant–animal interactions in order to assess the functional contributions of these structures. Results show that the nectary disc and petiolar nectaries are complex working units consisting of at least epidermis and ground tissue, while the other structures are glandular trichomes. Various groups of metabolites (lipids, phenolic compounds, polysaccharides, terpenoids, flavonoids, and alkaloids) were detected in all structures, while starch grains were only found in the nectary disc, petiolar nectaries, and their adjacent tissues. Integrating preliminary observation of animal visitors with micromorphological, anatomical, and histochemical results, two hypotheses are proposed: (I) nectary disc and staminal hairs are important for pollination as they potentially attract and reward floral visitors, and (II) petiolar nectaries, calycinal glands, and foliar glands contribute to plant defense. Specifically, petiolar nectaries and calycinal glands provide protection from herbivores via guard ants, while calycinal and foliar glands may use plant metabolites to help prevent tissue damage from dehydration and insolation.

## 1. Introduction

Plants have evolved remarkable adaptations that allow them to tolerate harsh environmental conditions [1,2] and to shape their interactions with other organisms, whether it be communicating with other plants in the community [3], attracting mutualistic partners such as pollinators [4,5,6], or deterring antagonistic individuals such as herbivores [7,8]. Many of these adaptations involve metabolites that are delivered through diverse secretory structures, such as nectaries, stinging hairs, and osmophores [9,10,11,12]. In general, secretion refers to the release of substances from the protoplast [13] and sometimes includes localization of substances within the cell [14]. Plants differentiate their organs both internally and externally to form secretory structures, which are involved in various activities such as excess ion elimination, metabolic waste compartmentalization, pollinator attraction, and defense against herbivory [15]. Secretory structures are diverse in form, ranging from simple to complex working units, based upon their functions, secreted substances, and locations on the plant [13,14,16].

Convolvulaceae is a diverse family distributed mainly in tropical and warm temperate climates [17], and secretory structures have been examined in several species within the family. In particular, two internal secretory organs have been reported, laticifers and crystal idioblasts, which possibly contribute to chemical defense and detoxification, respectively [18,19,20,21], and several other external secretory structures with different forms and functions, found on various plant parts, have also been reported. For example, peltate glands are ubiquitous on the epidermis of vegetative organs, with most reports coming from leaf investigation, and they are believed to contribute to protection from unsuitable environmental conditions and herbivory [18,20,22,23,24,25,26]. Moreover, glandular hairs on staminal filament bases have been found in several species within Convolvulaceae and are of taxonomic value to the family [27,28]. A recent study examining the pollination of *Argyreia siamensis* (Craib) Staples suggested that chemicals that accumulate in the hairs might contribute to pollinator attraction [29]. Nectaries are another common external secretory structure and can be found in three locations: flower, receptacle (or sometimes defined as part of the calyx or pedicel), and petiole. The role of the floral nectary (nectary disc) in rewarding pollinators has been well-established, and most species in the family are partially or completely dependent on pollinators [29,30,31,32]. While the floral nectary is conserved throughout the family, nectaries on petioles and/or receptacles predominantly occur in *Ipomoea* L., *Rivea* Choisy, and a few species from *Decalobanthus* Ooststr. and *Cuscuta* L. Extrafloral nectaries are generally considered to contribute to plant defense by attracting guard insects, which have mostly been reported to be ants inhabiting the surrounding areas [33,34,35,36,37,38].

One species in the Convolvulaceae family with unique secretory structures, such as prominent petiolar nectaries, is *Rivea ornata* (Roxb.) Choisy. *Rivea ornata* is one out of just three species from the genus *Rivea* Choisy, and its distribution ranges from the Indian subcontinent across the Eastern Himalaya to Indochina, but it is only rarely found in Thailand [39]. Most descriptions of this species mention the occurrence of a nectary disc and petiolar nectaries [17,28,40,41]. Additional observations of dried herbaria specimens and living plants in field surveys revealed the noticeably more prominent petiolar nectaries of *R. ornata*, as compared to related species, making it taxonomically significant and also leading to questions about the functional significance of such prominent extrafloral nectaries. Examination of the entire plant revealed that this species possesses numerous types of external secretory organs, in addition to petiolar nectaries. However, there is limited micromorphological, anatomical, and histochemical evidence to explain or provide support for their structures and functions.

Therefore, the aim of this study was to examine five external secretory structures in *R. ornata*, i.e., the nectary disc, petiolar nectaries, calycinal glands, staminal hairs, and foliar glands, in order to (I) identify and describe their micromorphological and anatomical features, (II) detect the main classes of chemicals in the accumulated substances and tissues by histochemical methods, and (III) conduct preliminary observations of animals visiting these secretory structures. We predicted that the diverse external secretory structures of *R. ornata* help shape their interactions with various, mainly insect, species (ranging from mutualistic to antagonistic), and use micromorphological, anatomical, histochemical, and ecological data to formulate hypotheses about these interactions.

## 2. Materials and Methods

### 2.1. Study Species and Sample Collection

**Figure 1:**
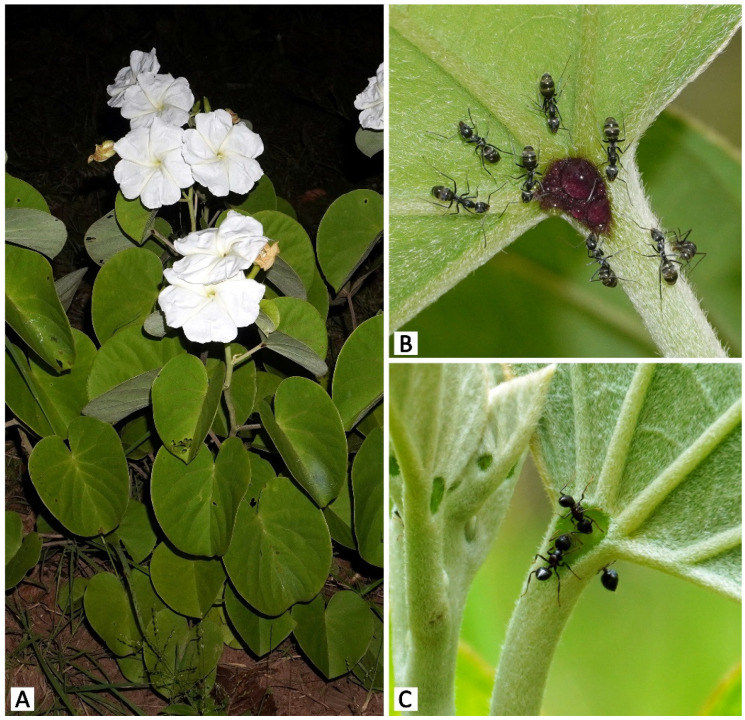
Photos of *Rivea ornata* from the two study populations. (**A**) A *Rivea ornata* plant during the flowering season. (**B**) A dark purple petiolar nectary at the Mae Hong Son study site surrounded by *Camponotus rufoglaucus* ants foraging on the droplet of nectar observed in the center of the nectary. (**C**) A green petiolar nectary at the Sakon Nakhon study site visited by *Crematogaster* sp. ants foraging on nectar.

*Rivea ornata* (Roxb.) Choisy is a perennial shrub with woody rootstock predominantly found in the understory of deciduous dipterocarp forests and mixed forests [28]. Flowers are fragrant, white, salverform, and night-blooming [28]. Anthesis usually starts around 1800 h, shortly before sunset (1830–1900 h), and lasts throughout the night until sunrise (0600 h) (NC, pers. obs.). The total accumulated nectar of a single flower ranges between 14–64 μL, and nectar sugar concentration tested using a handheld refractometer (Atago N1, 0–32%) ranges between 12–20% sucrose wt/wt (NC, pers. obs.). Flowering usually occurs during the rainy season, from around August to October [28] (NC, pers. obs.). Leaves are cordate and possess a pair of prominent glands at the apex of the petiole, widely known among convolvulaceous plants as “petiolar nectaries” [28,33,35,42,43]. Fruits are dry and horizontally dehiscent when mature, and contain four seeds embedded in the spongy matrix [28,39]. In Thailand, the current number of known *R. ornata* populations appears to be lower than the number of populations observed in herbarium records, which are mostly from the last 30–60 years, possibly due to deforestation and climate change. Plant materials used in this study were collected from the two largest known populations. The first study population (>30 individuals) was in Mae Hong Son province in northern Thailand, at an altitude of 590 m a.s.l. A second study population (ca. 30 individuals) was located in Sakon Nakhon province in northeastern Thailand, at an altitude of ca. 300 m a.s.l. Interestingly, there was a striking morphological difference found in plants between the two sites. Plants from the northern population displayed dark purple petiolar nectaries, while petiolar nectaries of plants in the northeastern population were green (Figure 1). Voucher specimens (northern population: *NC and PT 45, 63*; northeastern population: *NC and PT 60*) were prepared following the standard method for plant taxonomy [44] and deposited at the Forest Herbarium in Bangkok, Thailand (BKF). Fresh materials (including fully opened flowers and mature leaves) kept at 4 °C were used for histochemical examination, and materials fixed in 70% ethanol with a few drops of glycerol were used for micromorphological and anatomical study.

### 2.2. Micromorphology and Anatomy

Anatomical investigation by paraffin technique modified from Johansen [45] was carried out to examine the nectary disc, petiolar nectaries, calycinal glands, and foliar glands, using three to six flowers (nectary disc and calycinal glands) or leaves (petiolar nectaries and foliar glands) from different individuals from each population. Staminal hairs were excluded from anatomical investigation as it was difficult to obtain clear sections due to the disorganized nature of the hairs. For the four external secretory structures that could be sectioned, samples were dehydrated through a series of ethanol and butanol, embedded in paraffin, and cut using a sliding microtome (Leica SM2000R, Nussloch, Germany). The thin sections were affixed on glass slides with gelatin; deparaffinized by a series of xylene and ethanol; stained with a mixture of alcian blue, basic fuchsin, and acriflavine (AFA); and permanently mounted with DPX (distyrene, plasticizer, and xylene). The slides were examined and photographed using an Olympus CX21 light microscope equipped with a Sony α6400 digital camera.

To examine the surface of the secretory structures, peeled epidermal layers with AFA staining were prepared to observe both sides of the leaf lamina (to examine foliar glands), the petiolar nectaries, and the adaxial sepal surfaces (to examine calycinal glands). To examine staminal hairs, we performed a clearing technique using a potassium hydroxide solution and Clorox with toluidine blue O staining. These samples were temporally mounted in water and observed using the same microscope setup as described in the previous paragraph. Additionally, nectary discs were dehydrated using an acetone series, critical-point dried (Hitachi HCP-2, Macquarie Park, NSW, Australia), coated with platinum–palladium using an ion sputter (Hitachi E-102), and observed under a scanning electron microscope (SEM) (Hitachi S-2500). External secretory structures were described using anatomical terminology following Metcalfe and Chalk [18], Werker [46], and Evert [13].

### 2.3. Histochemistry

Thin sections of fresh materials were obtained from nectary discs, petiolar nectaries, sepals for calycinal glands, and leaves for foliar glands using a sliding microtome (Leica SM2000R), and staminal hairs were pulled out from the filaments. Five flowers and leaves from each population were used for each test, with three to five repetitions per flower or per leaf to confirm the results. The samples were treated with histochemical assays as follows: Sudan black B [47] and neutral red [48] for total lipids, ferric chloride [45] and potassium dichromate [49] for general phenolic compounds, Lugol’s iodine [45] for starch, periodic acid–Schiffs’s reagent (PAS) [50,51] for neutral polysaccharides, ruthenium red [45] for acidic polysaccharides, mercuric bromophenol blue [52] for proteins, Nadi reagent [53] for terpenoids, Naturstoff reagent A [48] for flavonoids, and Dragendorff [54] and Wagner’s [55] reagents for alkaloids. Neutral red and Naturstoff treatments were observed using a fluorescence microscope (Olympus BX53 with a DP73 camera set, Waltham, MA, USA) with 365 nm and 436 nm exciter filters, respectively, while the remaining treatments were observed under a light microscope (Olympus CX21 equipped with a Sony α6400 digital camera, Tokyo, Japan). Additionally, UV autofluorescence of the tissues were observed in each structure in general.

### 2.4. Preliminary Observation of Rivea ornata Visitors

Opportunistic observations were conducted to examine the overall species diversity of animal visitors (i.e., we did not quantify visitation frequency). Only visitors that appeared to interact with the examined secretory structures were recorded, and we categorized their potential role based on their behavior with *R. ornata*. The categories were: herbivore (consumes plant organs, such as leaves or corolla lobes), potential protector (consumes exudates and may protect plant from herbivores), exudate consumer (consumes exudates but likely does not offer protection from herbivores), and potential pollinator (visits flowers and possibly pollinates them). An animal visitor could be classified under more than one category, with the exception of the potential protector and exudate consumer categories, which were mutually exclusive. The Mae Hong Son population was examined during 14–16 September 2019 and 15–19 September 2020, and the Sakon Nakhon population was examined during 21–22 September 2019 and 7–11 September 2020. Direct observations were performed between 600–2200 h to cover both diurnal and nocturnal visitors as much as possible given field constraints, accounting for 18 h in 2019 and 35 h in 2020 for the Mae Hong Son study site, and for 8 h in 2019 and 30 h in 2020 for the Sakon Nakhon study site. Animal visitors were photographed, and some were collected and anesthetized in ethyl acetate vapor. Identification was made to the lowest taxonomic rank possible using various identification guides, e.g., *Ants of Thailand* [56] and *Thailand butterfly guide* [57], as well as consulting with entomologists (see Acknowledgements). Additionally, in 2020, action cameras (Yi Lite, Xiaomi) were set up to continuously record crepuscular and nocturnal visitor behavior between 600–1800 h, accounting for 60 and 48 h at the Mae Hong Son and Sakon Nakhon study sites, respectively. Since the action cameras were not equipped with night vision, plants were dimly illuminated with red light. Due to the relatively low resolution of the video footage, animal visitors were classified to the level of order.

## 3. Results

### 3.1. Micromorphology and Anatomy

**Figure 2:**
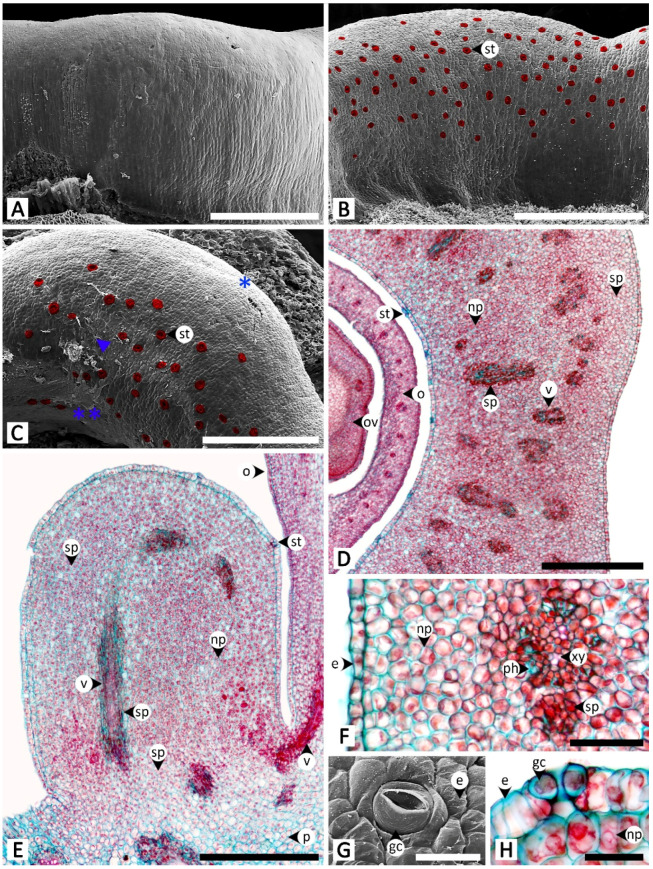
Micromorphology and anatomy of the nectary disc of *Rivea ornata*. (**A**) Abaxial surface without nectarostomata. (**B**) Adaxial surface showing the distribution of nectarostomata (highlighted in red). (**C**) Top view of the nectary disc showing nectarostomata (highlighted in red) located on the apical region and down along the adaxial side (double asterisk) but absent from the abaxial side (single asterisk), with trace amounts of exudate (arrowhead). (**D**) Transversal and (**E**) longitudinal views showing the epidermis, nectariferous parenchyma, subnectariferous parenchyma, and vascular tissues. (**F**) Close-up view of (**D**) from an area near the adaxial surface. (**G**) Surface image of nectarostoma. (**H**) Transversal section of nectarostoma. Note: (**A**–**C**,**G**) were taken with a scanning electron microscope, while (**D**–**F**,**H)** were taken with a light microscope. Abbreviations: e, epidermal cell; gc, guard cell; np, nectariferous parenchyma; o, ovary; ov, ovule; p, parenchyma; ph, phloem; sp, subnectariferous parenchyma; st, stoma; v, vascular tissue; xy, xylem. Scale bars: (**A**–**C**) 500 μm; (**D**,**E**) 1000 μm; (**F**) 250 μm; (**G**,**H**) 50 μm.

**Figure 3:**
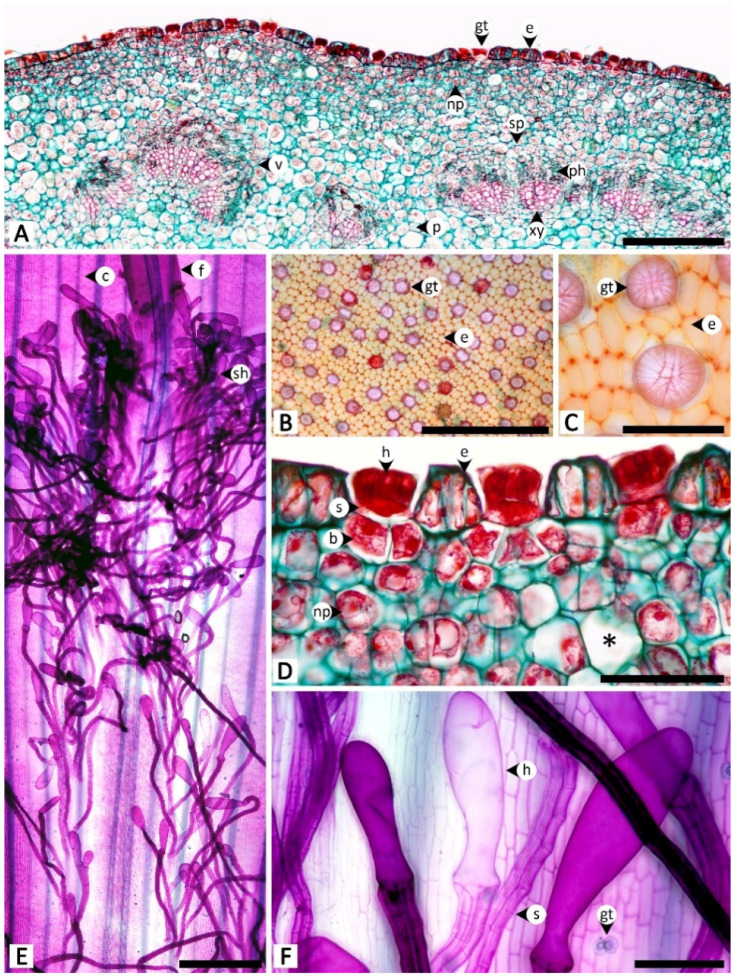
Micromorphology and anatomy of petiolar nectaries and staminal hairs of *Rivea ornata* via light microscopy. (**A**) Transversal view of petiole showing a petiolar nectary formed by epidermis, peltate glandular trichomes, nectariferous parenchyma, and subnectariferous parenchyma, as well as regions of petiole vascular tissues and cortex. (**B**) Surface of a petiolar nectary with peltate glandular trichomes distributed solitarily and evenly across the epidermis. (**C**) Close-up view of (**B**) showing details of the multicellular head of the peltate trichomes. (**D**) Close-up view of (**A**) along the epidermis showing details of the peltate trichomes, epidermal cells, nectariferous parenchyma, and a cell used to store druse crystals (asterisk). (**E**) The base of a stamen where it connects to the corolla tube covered in staminal hairs. (**F**) Details of staminal hairs showing apical glands and stalks. Abbreviations: b, base; c, corolla; e, epidermal cell; f, filament; gt, glandular trichome; h, head; np, nectariferous parenchyma; p, parenchyma; ph, phloem; s, stalk; sh, staminal hair; sp, subnectariferous parenchyma; v, vascular tissue; xy, xylem. Scale bars: (**A**,**E**) 1000 μm; (**B**) 500 μm; (**C**,**D**) 100 μm; (**F**) 200 μm.

**Figure 4:**
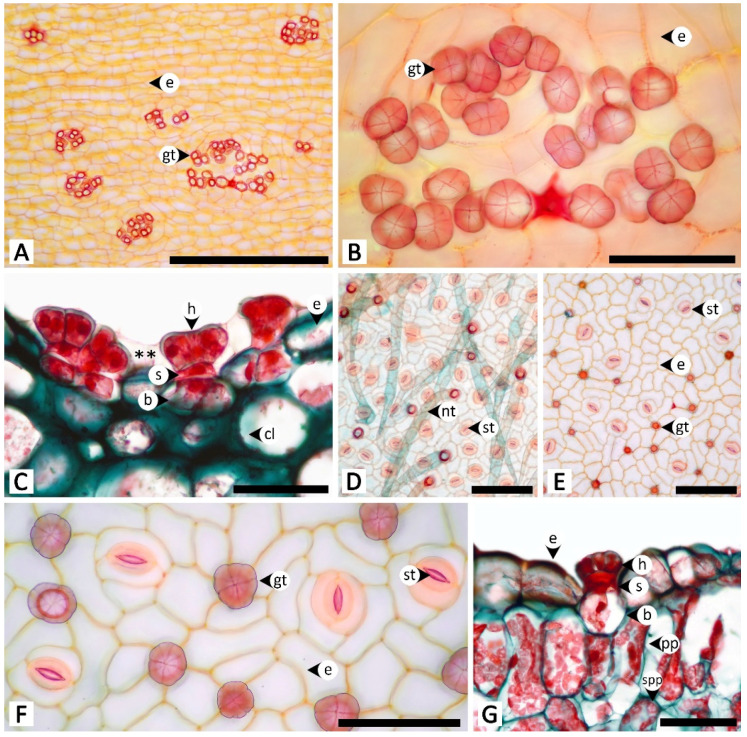
Micromorphology and anatomy of calycinal (**A**–**C**) and foliar (**D**–**G**) glands of *Rivea ornata* via light microscopy. (**A**) Adaxial surface of a sepal with clusters of calycinal glands. (**B**) Close-up view of (**A**) showing details of the multicellular head of the calycinal glands. (**C**) Transversal view of the adaxial side of a sepal showing a cluster of calycinal glands in the shallow pit with traces of exudate (double asterisk). (**D**) Abaxial leaf surface possessing only non-glandular trichomes. (**E**) Adaxial leaf surface possessing only glandular trichomes distributed solitarily on the epidermis. (**F**) Close-up view of (**E**) showing details of the multicellular head of the glandular trichomes. (**G**) Transversal view of a leaf showing details of the glandular trichomes. Abbreviations: b, base; cl, collenchyma; e, epidermal cell; f, filament; gt, glandular trichome; h, head; nt, non-glandular trichome; pp, palisade parenchyma; s, stalk; spp, spongy parenchyma; st, stoma. Scale bars: (**A**) 500 μm; (**B**,**F**) 100 μm; (**D**,**E**) 200 μm; (**G**) 50 μm.

Sectional and epidermal examination revealed that the nectary disc and petiolar nectaries function as complex working units formed by at least epidermal tissue and ground tissue (and vascular tissue is also found in nectary discs), while the calycinal glands, staminal hairs, and foliar glands are glandular trichomes formed from modified epidermal tissue only (Figure 2, Figure 3 and Figure 4).

The nectary disc is a rounded, pentagonal, bulging ring that is slightly concave planar along the sides, embracing around a quarter of the height of the ovary from where the nectary disc attaches to the receptacle. Scanning electron microscopy revealed that surfaces are glabrous (Figure 2A–C). Permanently opened nectarostomata were found on the apical and adaxial regions of the nectary disc (Figure 2B,C,G,H). Transversal and longitudinal sections revealed that the nectary disc is composed of three regions (epidermis, nectariferous parenchyma, and subnectariferous parenchyma) and is vascularized (Figure 2D–F). The nectary epidermal cells are anticlinally arranged in a single-cell layer. The nectariferous parenchyma consists of unorganized isodiametric cells with thin walls and dense cytoplasmic content, located underneath the epidermis. Vascular bundles pass through the middle region of the nectariferous parenchyma parallel to the longitudinal section outline, connecting to the vascular system in the receptacle and ovary (Figure 2D,E). Two types of subnectariferous parenchyma cells were found. The first type consists of elongated cells with intensely stained cytoplasm that are smaller in diameter than the nectariferous parenchyma cells and are arranged in parallel surrounding the vascular tissue (Figure 2D–F). The second type consists of cells that, compared to the nectariferous parenchyma cells, are larger in size, have looser cytoplasmic components, and are located in the area where the nectary disc attaches to the receptacle (Figure 2E).

The petiolar nectaries, in transversal view, appeared as a working unit comprising peltate trichomes on the surface and nectariferous tissue beneath (Figure 3A,D). The single-cell layer of the petiolar nectary epidermis is different from the adjacent areas by having anticlinal elongated epidermal cells. When viewed from the top, peltate trichomes are distributed solitarily and evenly across the petiolar nectary epidermis (Figure 3B,C). The trichomes are composed of three parts: an asymmetrical, radially divided, multicellular head; a unicellular stalk; and a uni- to multicellular base (Figure 3C,D). Only the basal cells penetrate into the nectariferous parenchyma; the head and stalk cells are level with the epidermal cells, or slightly sunken in the epidermal layer (Figure 3D). The nectariferous parenchyma cells of petiolar nectaries are polyhedral with dense cytoplasm (Figure 3A,D). Subnectariferous parenchyma cells are larger in size and have less cytoplasmic content than the nectariferous parenchyma cells. They span from the nectariferous parenchyma to the cortex zone, with several areas connecting to vascular bundles (Figure 3A). In the fresh sample sections, druse crystals were observed in nectariferous tissue cells but they vanished during the process of making permanent slides, thus leaving only large parenchyma cells lacking cytoplasmic content (Figure 3D).

Staminal hairs are distributed densely at the base of each filament (where they attach to the corolla), as well as along the adjacent areas between each filament base on the inner side of the corolla (Figure 3E). Two parts of the staminal hairs are noticeably distinct, i.e., head and stalk, but basal cells could not be differentiated from the other epidermal cells (Figure 3E,F). The head is unicellular and obovoid or pyriform. The stalk is cylindrical and formed by rows of long cells (Figure 3E,F). Peltate glandular trichomes were also sparsely detected among the hairs located on the inner corolla (Figure 3E).

Calycinal glands are dispersed across the epidermis of the adaxial surface of sepals, in the form of solitary peltate trichomes, or in clusters of up to 30, and nested in a shallow pit on the surface (Figure 4A–C). The trichomes contain three parts: a radially divided, eight-cell head; a unicellular stalk; and a one- to few-cell base (Figure 4B,C). Tissues under the glands were found to be collenchyma with sparse cell content (Figure 4C).

Foliar glands were found only on the adaxial surface of leaf blades, scattered solitarily and evenly on the epidermis among the stomata, while all trichomes on the opposite side of the leaf surface were non-glandular (Figure 4D,E). Similar to the structure of calycinal glands, the glandular trichomes of foliar glands are peltate (Figure 4E,F), containing three parts: a radially divided, eight-cell head; a unicellular stalk; and a unicellular base (Figure 4F,G). Transversal view showed that the peltate trichomes are slightly sunken, so as to have the same height as the surrounding epidermal cells (Figure 4G). A part of the basal cell extends into the palisade mesophyll (Figure 4G).

### 3.2. Histochemistry

**Figure 5:**
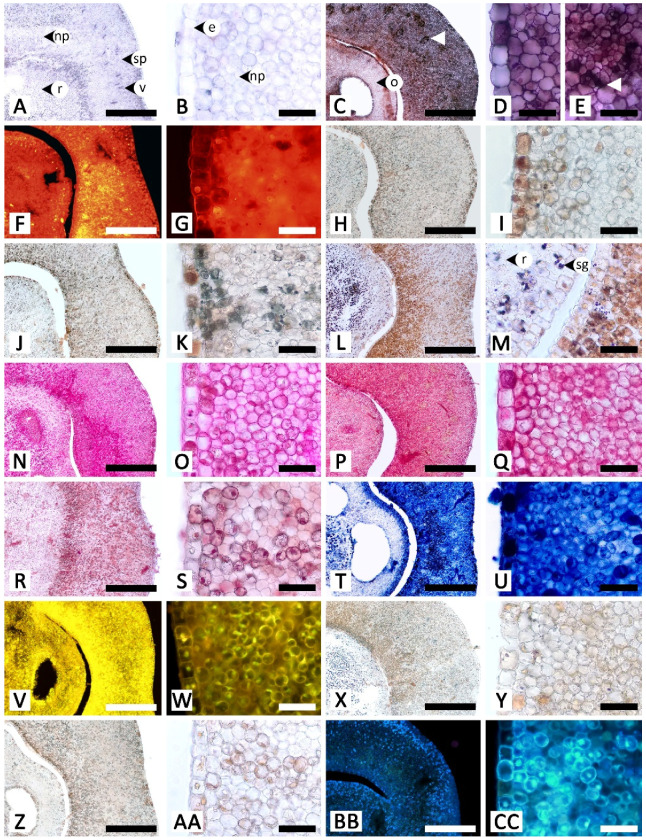
Histochemical results obtained from the nectary disc of *Rivea ornata*. (**A**) Transversal view and (**B**) close-up view of an unstained nectary disc. (**C**–**E**) Sudan black B test for total lipids; note cells in the subnectariferous parenchyma presenting intense positive results in accumulated substances (arrowhead). (**F**,**G**) Neutral red fluorochrome test for total lipids. (**H**,**I**) Ferric chloride test for phenolic compounds. (**J**,**K**) Potassium dichromate test for phenolic compounds. (**L**,**M**) Lugol’s iodine test for starch; note the positive results also present in parenchyma tissue of the receptacle. (**N**,**O**) Periodic acid–Schiff’s reagent (PAS) test for neutral polysaccharides. (**P**,**Q**) Ruthenium red test for acidic polysaccharides. (**R**,**S**) Mercuric bromophenol blue test for proteins. (**T**,**U**) Nadi reagent test for terpenoids. (**V**,**W**) Naturstoff reagent A under fluorescent microscopy detecting flavonoids. (**X**,**Y**) Dragendorff reagent test for alkaloids. (**Z**,**AA**) Wagner’s reagent test for alkaloids. (**BB**,**CC**) UV autofluorescence of the nectary disc. Note: (**C**–**Q**,**T**–**AA**) show positive reactions, while (**R**,**S)** show negative reactions. Abbreviations: e, epidermal cell; np, nectariferous parenchyma; o, ovary; r, receptacle; sg; starch grain; sp, subnectariferous parenchyma; v, vascular tissue. Scale bars: (**A**,**C**,**F**,**H**,**J**,**L**,**N**,**P**,**R**,**T**,**V**,**X**,**Z**,**BB**) 500 μm; (**B**,**D**,**E**,**G**,**I**,**K**,**M**,**O**,**Q**,**S**,**U**,**W**,**Y**,**AA**,**CC**) 50 μm.

**Figure 6:**
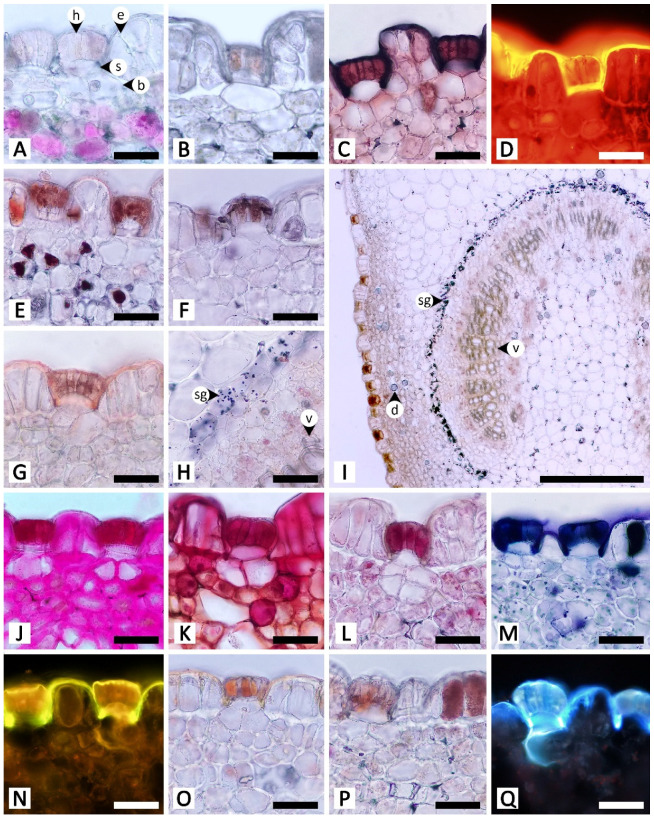
Histochemical results obtained from petiolar nectaries of *Rivea ornata*. (**A**) Unstained petiolar nectary from the Mae Hong Son study site showing anthocyanins in the nectariferous parenchyma. (**B**) Unstained petiolar nectary from the Sakon Nakhon study site without anthocyanins in the nectariferous parenchyma. (**C**) Sudan black B test for total lipids. (**D**) Neutral red fluorochrome test for total lipids. (**E**) Ferric chloride test for phenolic compounds. (**F**) Potassium dichromate test for phenolic compounds. (**G**–**I**) Lugol’s iodine test for starch; note that positive results are only present in a strand of parenchyma cells surrounding the vascular bundle. (**J**) Periodic acid–Schiff’s reagent (PAS) test for neutral polysaccharides. (**K**) Ruthenium red test for acidic polysaccharides. (**L**) Mercuric bromophenol blue test for proteins. (**M**) Nadi reagent test for terpenoids. (**N**) Naturstoff reagent A under fluorescent microscopy detecting flavonoids. (**O**) Dragendorff reagent test for alkaloids. (**P**) Wagner’s reagent test for alkaloids. (**Q**) UV autofluorescence of petiolar nectaries. Note: (**C**–**F**,**H**–**K**,**M**–**P**) show positive reactions, while (**G**,**L**) show negative reactions. Abbreviations: b, base; d, druse crystal; e, epidermal cell; h, head; s, stalk; sg, starch grain; v, vascular tissue. Scale bars: (**A**–**H**,**J**–**Q**) 50 μm; (**I**) 500 μm.

**Figure 7:**
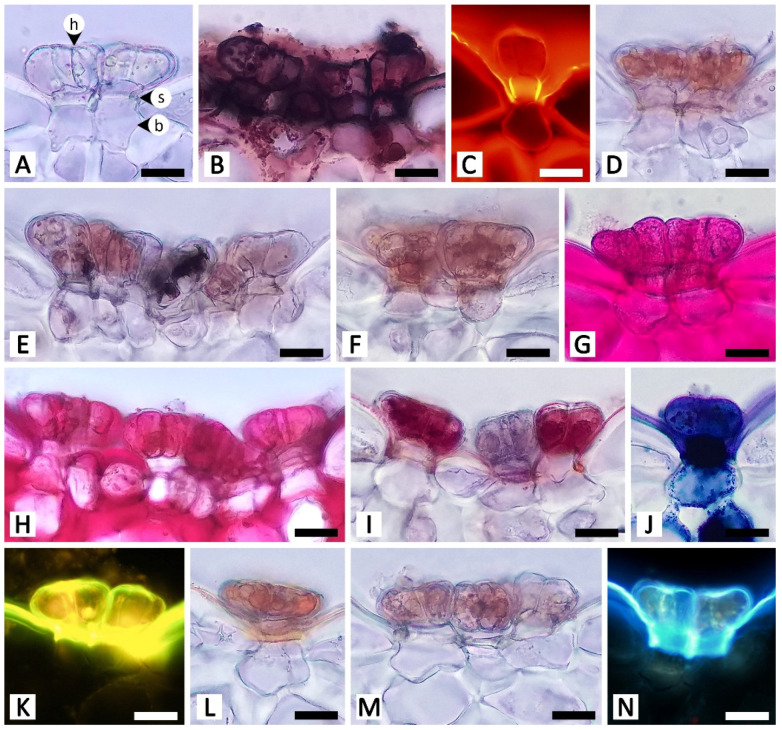
Histochemical results obtained from calycinal glands of *Rivea ornata*. (**A**) Unstained calycinal glands. (**B**) Sudan black B test for total lipids. (**C**) Neutral red fluorochrome test for total lipids. (**D**) Ferric chloride test for phenolic compounds. (**E**) Potassium dichromate test for phenolic compounds. (**F**) Lugol’s iodine test for starch. (**G**) Periodic acid–Schiff’s reagent (PAS) test for neutral polysaccharides. (**H**) Ruthenium red test for acidic polysaccharides. (**I**) Mercuric bromophenol blue test for proteins. (**J**) Nadi reagent test for terpenoids. (**K**) Naturstoff reagent A under fluorescent microscopy detecting flavonoids. (**L**) Dragendorff reagent test for alkaloids. (**M**) Wagner’s reagent test for alkaloids. (**N**) UV autofluorescence of calycinal glands. Note: (**B**–**E**,**G**,**H**,**J**–**M**) show positive reactions, while (**F**,**I**) show negative reactions. Abbreviations: b, base; h, head; s, stalk. Scale bars 20 μm.

**Figure 8:**
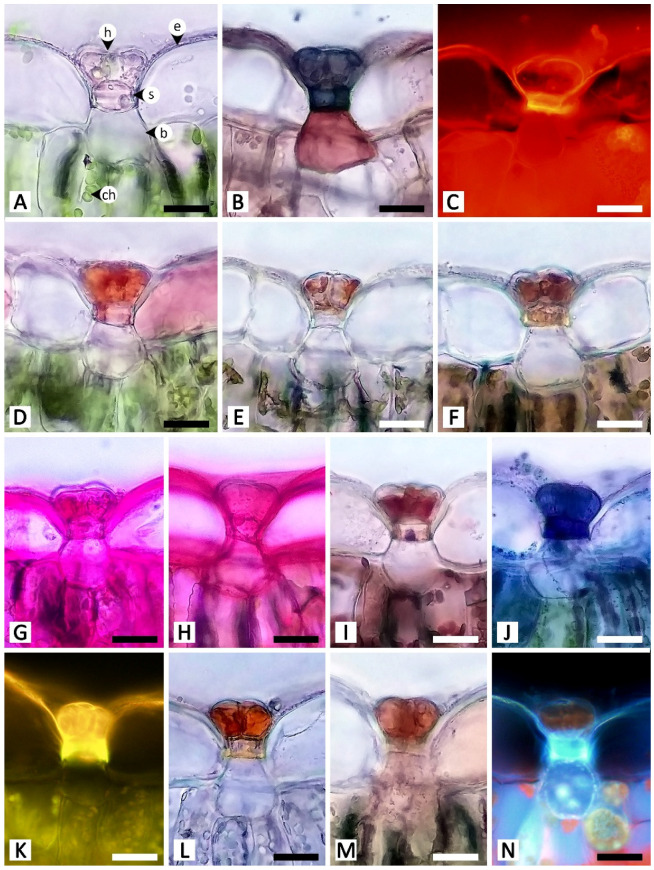
Histochemical results obtained from foliar glands of *Rivea ornata*. (**A**) Unstained foliar gland. (**B**) Sudan black B test for total lipids. (**C**) Neutral red fluorochrome test for total lipids. (**D**) Ferric chloride test for phenolic compounds. (**E**) Potassium dichromate test for phenolic compounds. (**F**) Lugol’s iodine test for starch. (**G**) Periodic acid–Schiff’s reagent (PAS) test for neutral polysaccharides. (**H**) Ruthenium red test for acidic polysaccharides. (**I**) Mercuric bromophenol blue test for proteins. (**J**) Nadi reagent test for terpenoids. (**K**) Naturstoff reagent A under fluorescent microscopy detecting flavonoids. (**L**) Dragendorff reagent test for alkaloids. (**M**) Wagner’s reagent test for alkaloids. (**N**) UV autofluorescence of foliar gland. Note: (**B**–**E**,**G**,**H**,**J**–**M**) show positive reactions, while (**F**,**I**) show negative reactions. Abbreviations: ch, chloroplast; b, base; e, epidermal cell; h, head; s, stalk. Scale bars 20 μm.

**Figure 9:**
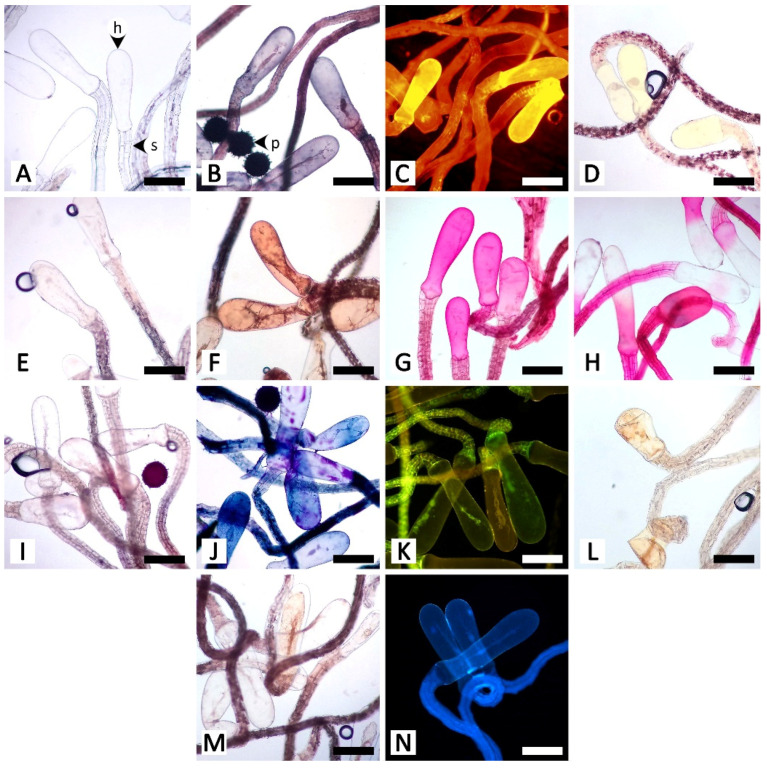
Histochemical results obtained from staminal hairs of *Rivea ornata*. (**A**) Unstained staminal hairs. (**B**) Sudan black B test for total lipids. (**C**) Neutral red fluorochrome test for total lipids. (**D**) Ferric chloride test for phenolic compounds. (**E**) Potassium dichromate test for phenolic compounds. (**F**) Lugol’s iodine test for starch. (**G**) Periodic acid–Schiff’s reagent (PAS) test for neutral polysaccharides. (**H**) Ruthenium red test for acidic polysaccharides. (**I**) Mercuric bromophenol blue test for proteins. (**J**) Nadi reagent test for terpenoids. (**K**) Naturstoff reagent A under fluorescent microscopy detecting flavonoids. (**L**) Dragendorff reagent test for alkaloids. (**M**) Wagner’s reagent test for alkaloids. (**N**) UV autofluorescence of staminal hairs. Note: (**B**–**E**,**G**,**H**,**J**–**M**) show positive reactions, while (**F**,**I**) show negative reactions. Abbreviations: h, head; p, pollen grain; s, stalk. Scale bars 200 μm.

The histochemical assays revealed that the secretory structures in *R. ornata* produce various groups of metabolites, as all examined compounds tested positive, with the exception of proteins (Table 1 and Figure 5, Figure 6, Figure 7, Figure 8 and Figure 9). Lipids appear to be restricted to structural layers such as cell walls, while phenolic compounds, terpenoids, flavonoids, and alkaloids are mainly found in substances that accumulate in cytoplasmic components. Polysaccharides, in general, occur in both cell walls and cell contents (Table 1 and Figure 5, Figure 6, Figure 7, Figure 8 and Figure 9). Additionally, starch grains were found only in the nectary disc, and only sparsely (Table 1 and Figure 5L,M), but they were also detected in abundance in parenchyma cells of the receptacle (Figure 5L,M) and in cells near petiolar nectaries (Figure 6H,I).

The nectary disc contained all classes of chemicals tested in this study except proteins. It was the only structure that produced starch in its tissues. The histochemical results from the nectary epidermis and the nectariferous tissue of the nectary disc were congruent (Table 1 and Figure 5). However, the positive results for most of the detected chemicals were more pronounced in the nectariferous parenchyma near the adaxial epidermis (e.g., Figure 5F,N,P,T,V,X). Lipids appeared concentrated in the subnectariferous parenchyma cells surrounding vascular bundles (Figure 5C,E). Blue-green autofluorescence under UV wavelengths was also mainly found in cells in the abaxial regions (Figure 5BB,CC).

The unstained petiolar nectary sections revealed that the purple color observed in the Mae Hong Son population was caused by the accumulation of anthocyanin in the nectariferous parenchyma of the petiolar nectaries; anthocyanin was absent from the Sakon Nakhon population, resulting in green petiolar nectaries due to chloroplasts (Figure 6A,B). Histochemical results were not different between the two morphotypes. Substances with positive histochemical results were principally stored in the head of the glandular trichome and in the elongated epidermal cells of the petiolar nectaries, but they were sometimes also found in the stalk and basal cells of the glandular trichomes, or even in nectariferous cells (Table 1 and Figure 6). Starch grains were not found in the glandular trichomes, but accumulated in a strand of parenchyma cells located next to the outer edge of vascular bundles in petiolar nectaries (Figure 6H,I); some of the starch grains were located in the subnectariferous region, while others were found in the cortex, which was not a part of the secretory working unit in petiolar nectaries. The cuticle layer on glandular trichomes and epidermal cells exhibited blue autofluorescence under UV wavelengths (Figure 6Q).

Calycinal and foliar glands not only shared homologous structures but also presented similar histochemical results (Table 1; Figure 7 and Figure 8). Positive reactions were mainly found at the head and stalk cells of the trichomes. While lipids detected by Sudan black B were generally found in both head and stalk regions, the results of the neutral red assay revealed that lipids predominantly occurred in stalk cells (Figure 7B,C and Figure 8B,C). Terpenoids and flavonoids, as well as blue-green autofluorescence under UV wavelengths, were more pronounced in the stalk cells rather than the head cells (Figure 7J,K,N and Figure 8J,K,N). In addition to the vibrant blue-green autofluorescence, faint orangish-brown autofluorescence was observed for substances accumulated in the head of calycinal and foliar glands (Figure 7N and Figure 8N).

In the staminal hairs, positive histochemical results were found in both the head and stalk regions, but they generally showed different degrees of chromatic reaction (Table 1 and Figure 9). Only phenolic compounds were absent from the head of the hairs (Table 1 and Figure 9D,E). For lipids, while Sudan black B gave relatively indistinct positive reactions in both the head and stalk regions, neutral red fluorochrome results were noticeably evident at the head (Figure 9B,C). For neutral polysaccharides, the head stained magenta but the stalk stained red (Figure 9G). A similar result was observed for terpenoids, in which substances stored in the head turned violet or blue, but the wall of the head and the stalk were solely dark blue (Figure 9J). The test for acidic polysaccharides generally showed patterns of positive staining in the head, but some heads were completely stained, some were stained only at the area attaching to the stalk, and some were entirely unstained (Figure 9H). Both parts of the staminal hairs emitted dim blue autofluorescence under UV wavelengths (Figure 9N).

### 3.3. Preliminary Observations of Rivea ornata Visitors

**Figure 10:**
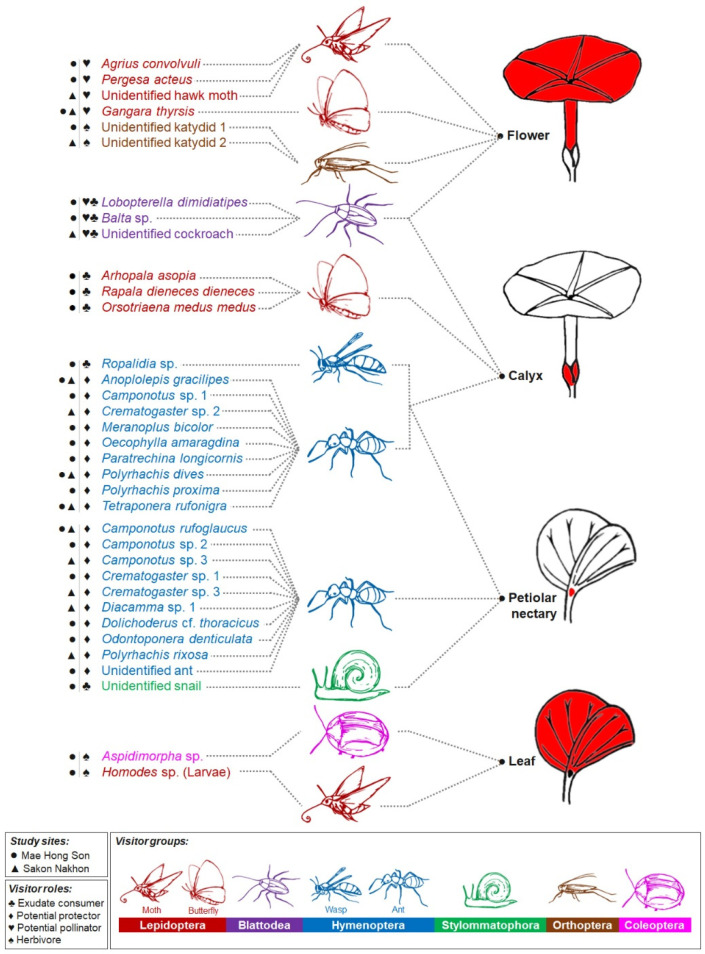
Diagram of animal visitors observed visiting plant parts where the studied external secretory structures are found (nectary disc and staminal hairs at flowers, calycinal glands at calyxes, petiolar nectaries at petioles, and foliar glands at leaves). Visitors were classified as: herbivore (consumes plant organs, such as leaves or flowers), potential protector (consumes exudates and may protect plant from herbivores), exudate consumer (consumes exudates but likely does not offer protection from herbivores), or potential pollinator (visits flowers and possibly pollinates them). Most visitors fell under a single category, with the exception of cockroaches (Blattodea), which were classified as both exudate consumers and potential pollinators. Note: Dashed lines connecting visitor names to plant parts only indicate that visits were observed, they do not indicate visitation frequency.

**Figure 11:**
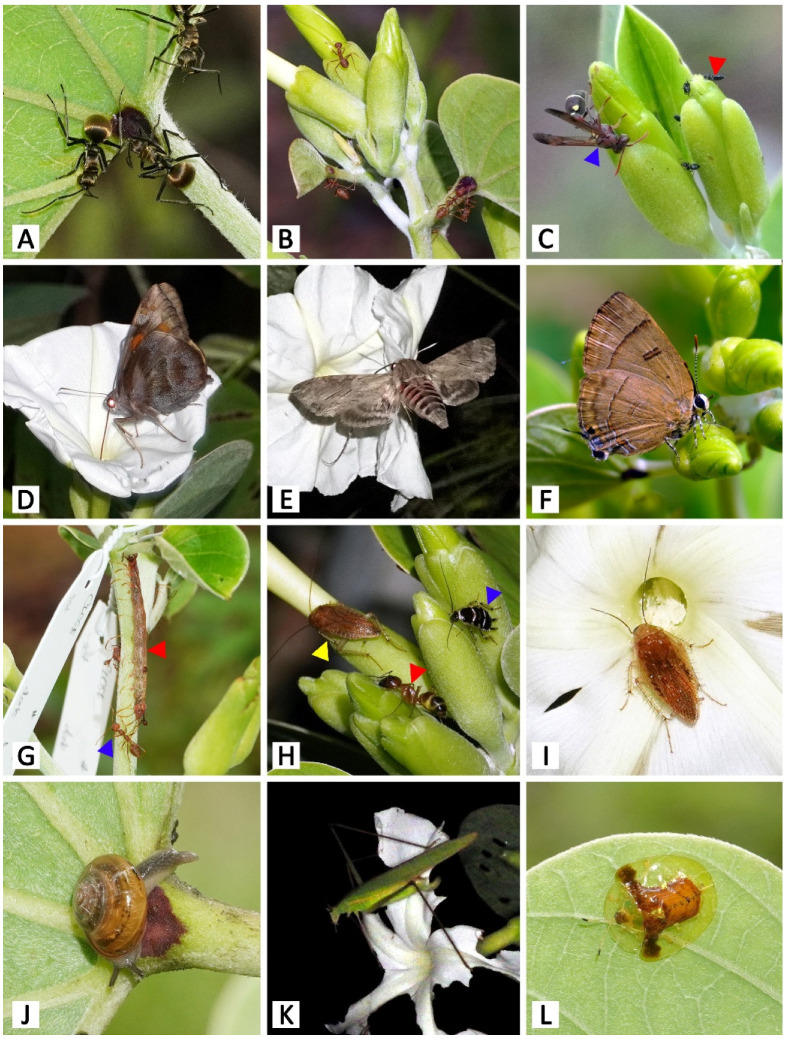
Animal visitors of *Rivea ornata* (hv: herbivore, pr: potential protector, ec: exudate consumer, po: potential pollinator). (**A**) *Polyrhachis proxima* (pr) on petiolar nectaries. (**B**) *Oecophylla amaragdina* (pr) on calyx and petiolar nectaries. (**C**) *Ropalidia* sp. (ec; blue arrowhead) and *Crematogaster* sp. (pr; red arrowhead) visiting calyces. (**D**) *Gangara thyrsis* (po) visiting a flower. (**E**) *Agrius convolvuli* (po) visiting a flower. (**F**) *Rapala dieneces dieneces* (ec) visiting a calyx. (**G**) Caterpillar of *Homodes* sp. (hv; red arrowhead) on a branch, undisturbed by *Oecophylla amaragdina* (pr; blue arrowhead). (**H**) *Balta* sp. (ec; yellow arrowhead), *Lobopterella dimidiatipes* (ec; blue arrowhead), and *Camponotus* sp. (pr; red arrowhead) on calyces. (**I**) *Balta* sp. (po) visiting a flower; note the pollen grains on its antennae. (**J**) Unidentified Helicinan snail (ec) on a petiolar nectary. (**K**) Unidentified katydid (hv) consuming the corolla of a flower. (**L**) *Aspidimorpha* sp. (hv) on a leaf.

Thirty-five taxa of insects and one Helicinan snail interacted with at least one of the four parts of the plant related to the studied secretory structures, i.e., calyx (calycinal glands), petioles (petiolar nectaries), flowers (nectary disc and staminal hairs), and leaf blades (foliar glands) (Figure 1B,C, Figure 10 and Figure 11). Among these visitors, 63% of all visitor diversity (22 taxa) were observed only at the Mae Hong Son study site, while 23% (8 taxa) were observed only at the Sakon Nakhon study site, and 14% (5 taxa) were found at both sites.

The majority of the visitors were hymenopterans, accounting for 57% of visitor species diversity. Within this order, we observed numerous ant species (Formicidae) (Figure 1B,C, Figure 10 and Figure 11A–C,G,H) and one paper wasp species (*Ropalidia* sp., Vespidae) (Figure 10 and Figure 11C), all of which visited either the petiolar nectaries alone or visited both the petiolar nectaries and calyxes. The wasps were active during the day and roamed in groups of up to approximately five individuals per plant. Ants were observed interacting with *R. ornata* at all times of day and night, patrolling mainly around the stems, petiolar nectaries, and calyxes (of both flower buds and mature, open flowers), where they made routes from the ground to find food, but they were not often observed on leaf blades. They were more often observed on young and newly mature leaves than on aged leaves. In general, ants occupying the same area of the plant (e.g., a branch containing inflorescences and leaves), or the entire plant, belonged to the same species. We observed one instance of territory protection, where *Oecophylla smaragdina* ants were aggressive towards individuals of *Camponotus* sp. While ants were generally not aggressive towards insects from other families (e.g., cockroaches and butterflies) found on areas of the plant that were not patrolled by ants (Figure 11H), multiple ant species were observed to act aggressively towards cockroaches on calyxes and nearby areas (e.g., the corolla tube surface next to the sepal apexes). Such aggression was observed during both day and night, with the ants attacking and chasing any cockroaches encountered, causing the cockroaches to move to other, ant-free, areas. Herbivores on plant organs beyond the ant-patrolling areas (e.g., flowers and leaf blades) were not observed to be chased or harmed by the ants.

Eight taxa of lepidopterans, accounting for 23% of total visitor species, were spotted as visitors either at flowers, calyxes, or leaves (Figure 10 and Figure 11D–G). Skippers (*Gangara thyrsis*, Hesperiidae) visited flowers at dusk, as soon as the flowers began to bloom, and sphinx moths (Sphingidae) visited flowers after it was dark (Figure 10 and Figure 11D,E). All butterfly and moth species inserted their proboscises through the long corolla tube (and in doing so, through the clump of staminal hairs), to access nectar stored in the nectar chamber. In doing so, their proboscises were certain to contact anthers and stigmas, which were closely bundled together due to the narrow diameter of the corolla tube. We, therefore, classified skippers and sphinx moths as potential pollinators because pollen grains were observed on the proboscises of individuals visiting *R. ornata* flowers, which were confirmed to be the pollen of *R. ornata* by examining captured specimens. Three butterfly species (Lycaenidae and Nymphalidae) were also observed visiting calyxes in the early morning, and they used their proboscises to forage on sepal exudate and leachate, so we classified them as exudate consumers (Figure 10 and Figure 11F). The larval stage of a species of *Homodes* moth (Erebidae) was observed consuming the leaves (and, therefore, classified as an herbivore), and they were associated with and protected by *Oecophylla smaragdina* (Formicidae) (Figure 10 and Figure 11G).

Three taxa of cockroaches (Ectobiidae, Blattodea), accounting for 9% of all visitor species, were mainly observed as exudate consumers at calyxes (Figure 10 and Figure 11H,I). They were active beginning in the evening, throughout the night, and during the early morning hours before sunlight directly illuminated the plants. Moreover, they were also found to visit the corolla throat and contact stigmas and anthers, possibly to consume stigma fluid or pollen. We, therefore, also classified them as potential pollinators, since pollen grains were observed on their antennae, head, legs, and dorsal areas of the thorax and abdomen.

A Helicinan snail (Stylommatophora, Helicoidea), kattydids (Orthoptera, Tettigoniidae), and tortoise beetles (Coleoptera, Chrysomelidae), together accounting for 11% of the visitor species diversity, were classified as exudate consumers, floral herbivores, and foliar herbivores, respectively (Figure 10 and Figure 11J–L). The snail visited petiolar nectaries where ants were not present (Figure 10 and Figure 11J). The katydids were active from night until the following morning; they only came during nights when the plants were in bloom, and they were observed consuming the corolla lobes of flowers, so that just the corolla tube and midpetaline bands remained (Figure 10 and Figure 11K). Tortoise beetles were frequently spotted consuming the leaf blades of young and newly mature leaves (Figure 10 and Figure 11L). We did not observe ants acting aggressively towards katydids and tortoise beetles.

## 4. Discussion

Our study revealed that diverse secretory organs can appear very similar micromorphologically and anatomically, such as calycinal and foliar glands, or have a completely distinct structure, such as the nectary disc. The richness of chemicals produced by these secretory structures, as evidenced by the histochemical tests performed, provide insight into their ecological roles, especially when assessed in combination with the visitor observation results. From our micromorphological, anatomical, histochemical, and ecological results, we propose two hypotheses, with support from the previous related literature, about the function of secretory organs in *Rivea ornata*: (I) the nectary disc and staminal hairs, which are floral secretory organs, are important in pollinator attraction [29,30], and (II) petiolar nectaries, calycinal glands, and foliar glands are associated with defense mechanisms. Specifically, petiolar nectaries and calycinal glands appear to provide indirect defense against herbivores by attracting guard ants [33,34,35,36,37], and the calycinal glands may also protect flowers and fruit from abiotic stressors [26,58]. Foliar glands appear to be related to a wide array of defensive mechanisms, e.g., reducing herbivory through secretion of plant metabolites or by harboring beneficial microbes, as well as providing protection against abiotic stress in hot and dry environments [19,25,36,59,60,61,62,63,64].

### 4.1. Floral Secretory Structures Potentially Important to Pollination

The micromorphological and anatomical features of the nectary disc found in *R. ornata* were generally similar to those of other species in the family, as described in previous studies, both in terms of nectary tissue composition and vascularization [29,30,65,66]. The secretion of nectar through modified stomata (i.e., nectarostomata) is common among flowering plants across diverse, taxonomically distant species [67]. Galetto and Bernardello [30] reported three species-specific distribution patterns for nectarostomata on the nectary disc epidermis in six species of *Ipomoea* L., i.e., homogeneously distributed over the surface, restricted to only the apex and base, and restricted only to the apical area. The latter distribution pattern was found in a related morning glory species, *Argyreia siamensis* (Craib) Staples [29]. Nectarostomata in *R. ornata*, however, presented a different pattern, appearing on the apical region and down along the adaxial surface of the epidermis, but absent from the abaxial surface. Nectarostomata are the openings through which nectar are secreted [68], and the function of the nectary disc in producing nectar as a reward for pollinators is widely acknowledged [30,69,70].

Several groups of metabolites detected in the tissues and accumulated substances of the nectary disc in *R. ornata* have also been found in the floral nectaries of other plant taxa, such as from Anacardiaceae, Solanaceae, *Zeyheria* Mart. (Bignoniaceae), and *Pedicularis* L. (Orobanchaceae) [71,72,73,74], and these studies often reported a lack of proteins in their nectariferous tissue as well. Starch grains present in the nectary disc and receptacle of *R. ornata* are likely the substrate that is subsequently hydrolyzed into sugars during the process of nectar production [75,76]. Polysaccharides and lipids stored in the cells could serve as sources of essential nectar nutrients for pollinators [67], and positive histochemical reactions for these two chemical classes also indicate their presence in the cell wall or cuticle layer, as they are primary components of these structures [77,78]. Moreover, the positive detection for lipids in cells within in the subnectariferous parenchyma of the nectary disc could indicate the presence of laticifers [36], however, they could not be identified with certainty using our anatomical technique. Tissues that appeared to be bundles of subnectariferous parenchyma and vascular tissue in *R. ornata* were also found and defined as secretory ducts in the nectary disc of *Argyreia siamensis* [29]. Alkaloids and phenolic compounds present in the nectary disc might serve to discourage visitors or herbivores that are susceptible to such chemicals [79,80,81]. Flavonoids are typically thought to be responsible for floral coloration to attract pollinators; in the case of *R. ornata*, since the nectary disc is hidden from pollinator view due to the narrow corolla tube, these chemicals may serve other functions, such as stress detoxification or defense against pathogens [82,83]. Terpenoids are crucial scent compounds that play prevalent roles in pollinator attraction [84] and in defense against herbivores [83]. The presence of terpenoids in the nectary disc tissues suggests that they may infuse into the nectar as well [85], however, the nectary disc is not the only organ producing scent in *R. ornata* flowers, since the staminal hairs also presented a strong positive histochemical reaction for terpenoids. Indeed, almost all of the metabolites found in the nectary disc were also found in the staminal hairs, which, given the fact that both occur in *R. ornata* flowers, suggests that these two organs function similarly and work synergistically.

Flowers of *R. ornata* closely match the typical sphingophilous pollination syndrome by having white, fragrant corollas with a long, narrow corolla tube and nocturnal anthesis [4]. As expected, sphingid moths visited *R. ornata* individuals with flowers and showed high potential to contribute to pollination, given that *R. ornata* pollen was found on their bodies. Pollinator observations made at *Ipomoea alba* L. flowers, a species with convergently evolved moth flowers similar to those of *R. ornata*, revealed that sphingid moths were the only pollinators of this species [30,31]. At night, white flowers exhibit the greatest contrast to dark backgrounds, and are perceived by moths via photoreceptors in their large, sensitive eyes [86], and, simultaneously, floral scents are detected by their antennae and help guide them to the flower [87,88]. Among the floral scent components known to attract moths, terpenoids, especially acyclic terpene alcohols such as linalool, nerolidol, and farnesol, are frequently among the most common [89,90,91]. Therefore, it is highly likely that the scent of *R. ornata* flowers stems from terpenoids produced in the nectary disc and staminal hairs. 

In addition to sphingid moths, we also observed two other insect groups visiting and potentially pollinating *R. ornata* flowers: skipper butterflies (Hesperiidae) and cockroaches (Blattodea). Although skipper butterflies are active during the day [92], they were able to visit *R. ornata* flowers during approximately the first hour of anthesis (1800–1900 h), before sunset. Pollination by cockroaches is relatively rare, with only 11 flowering plant species confirmed to date to have cockroaches as effective pollinators [4,93,94]. They do not have the long appendages necessary to forage on the nectar of *R. ornata*, given the long, narrow corolla tube, but the behaviors observed in this study correspond to previous reports that cockroaches feed on pollen and stigmatic exudates, and, thus, may help pollinate flowers [4,93]. We also observed pollen on the bodies of skipper butterflies and cockroaches, so further research is necessary to compare the contributions of sphingid moths, skipper butterflies, and cockroaches and to determine which groups are effective pollinators of *R. ornata*.

### 4.2. Secretory Structures Potentially Important to Defense

Petiolar nectaries are the only extrafloral nectary found in the genus *Rivea*, while several species of the sister genus *Ipomoea* exhibit either petiolar nectaries, receptacular nectaries, or both [33,34,35,36,37,38], and another genus in the family, *Cuscuta* L., is unique for nectaries located along the stem [95,96]. Based on the classification of petiolar nectaries studied in *Ipomoea* by Keeler and Kaul [35], the petiolar nectaries of *R. ornata* fits the description of a superficial nectary (secretory tissues located on the surface exposed to the environment), rather than one of the other two types, i.e., a crypt nectary (secretory tissues located deep within a cavity, with nectar transferred through a duct and released to the surface by a pore), or a basin nectary (secretory tissues located in slightly recessed depressions). Superficial nectaries are rare among *Ipomoea*, with the only other occurrence reported in *Ipomoea leptophylla* Torr. [42]. This type of nectary, however, also seems to occur in *Decalobanthus peltatus* (L.) A.R. Simões & Staples because its nectary appearance and tissue arrangement is similar to our findings in *R. ornata* [97]. The petiolar nectaries of *R. ornata* and other species in the family have similar peltate trichomes on the petiolar nectary epidermis and potentially secrete nectar through these trichomes as the main pathway, while petiolar nectaries in other plant lineages have been reported to secrete nectar through either a modified epidermal layer (e.g., *Sapium biglandulosum* Müll. Arg., Euphorbiaceae; *Smilax polyantha* Griseb., Smilacaceae; and *Passiflora* spp., Passifloraceae) or through various kinds of glandular trichomes (e.g., *Hibiscus forsteri* F. D. Wilson and *Eriotheca gracilipes* (K. Schum.) A. Robyns in the Malvaceae family) [36,43,44,45,46,47,48,49,50,51,52,53,54,55,56,57,58,59,60,61,62,63,64,65,66,67,68,69,70,71,72,73,74,75,76,77,78,79,80,81,82,83,84,85,86,87,88,89,90,91,92,93,94,95,96,97,98,99,100,101,102].

Our work revealed that tissues involved with the petiolar nectaries of *R. ornata* produced diverse groups of metabolites, similar to those found in the nectary disc, and they probably function in similar ways as discussed for the nectary disc. In contrast, Martins et al. [36] reported histochemical tests of the crypt petiolar nectaries in *Ipomoea asarifolia* Roem. & Schult. and found positive reactions only for polysaccharides. The presence of chemicals involved in defense (such as alkaloids, phenolic compounds, flavonoids, and terpenoids) in *R. ornata* petiolar nectaries might be due to their exposed nature, which poses a higher risk of getting damaged from pathogens, desiccation, and UV radiation, therefore rendering protective chemicals essential, as opposed to *I. asarifolia* petiolar nectaries, which are hidden [36,43]. Even though the boundary of the petiolar nectaries defined in this study was limited to the secreting tissues near the surface, localizations of starch grains near these secreting tissues (presumably as a precursor of nectar sugars) imply that the areas where nectar production occurs are not limited to just the secreting tissues [75,76]. The petiolar nectaries of the northeast population lacked anthocyanins, resulting in green petiolar nectaries, which possibly have a genetic basis, such as decreased expression of genes related to anthocyanin biosynthesis [103,104]. Anthocyanins belong to a class of flavonoids that are widely known to be involved in plant response to both biotic and abiotic stressors [105,106]. Thus, further work is needed to assess how the lack of this protective substance in some populations of *R. ornata* affects plant fitness. 

The calycinal glands found in *R. ornata* were morphologically identical to the calycinal glands of other species in the family [26,58]. *Stictocardia tiliifolia* (Desr.) Hallier f., *Operculina turpethum* (L.) Silva Manso, and *O. codonantha* (Benth.) Hallier f. were reported to secrete obvious quantities of slimy fluids, while exudates from other species, e.g., *Ipomoea pes-caprae* (L.) R.Br., *I. quamoclit* L., and *Argyreia mollis* (Burm.f.) Choisy, were secreted in unnoticeable amounts [58], and this latter case was also found for *R. ornata*. Nevertheless, the positive reaction of the ruthenium red test showed that the calycinal glands of *R. ornata* likely contain mucilaginous and hygroscopic substances, as was also found in *Ipomoea cairica* (L.) Sweet [26], and were defined as colleters (any of the morphologically diverse secretory organs functioning homologously in secreting mucilaginous or resinous fluids [13]). Based on the properties of the secreted substances, calycinal glands are believed to protect flower buds, from their development until fruiting, from desiccation and insolation [26,58,107].

Although the petiolar nectaries and calycinal glands of *R. ornata* are located on different parts of the plant, they appear to have the same function in feeding visitors, especially diverse species of ants. Ants are widely known to be the main nectar consumer of extrafloral nectaries and are generally acknowledged as guards against herbivores [9,108,109], but we have not found any reports about ants or other insects visiting secretory organs that function as colleters. Therefore, our study may be the first to report another potential role of calycinal glands, or calycinal colleters, beyond protection from abiotic factors. It is possible that *R. ornata* gained the capability of attracting guard ants through its calycinal colleters instead of developing receptacular nectaries, which are found in several *Ipomoea* species [35]. Nevertheless, the effectiveness of protection by guard ants depends on nectar quality and the natural aggressiveness of each ant species [110,111]. Our study further suggests that the effectiveness of ant guards also depends on the density of external secretory structures, the distribution of external secretory structures on the plant, and any plant traits that exclude ants (e.g., corolla tubes too narrow for ants to enter), as these traits influence the plant’s ability to attract ant guards to specific areas or prevent them from accessing certain areas. Unlike other plant species that have ant-attracting secretory structures distributed on the leaf blade and leaf margin or located on the exposed parts of the flower, such as *Passiflora* spp., *Miconia tococa* (Desr.) Michelang., *Turnera subulata* Sm., and *Mallotus japonicus* (L.f.) Müll.Arg. [109,112,113,114,115], petiolar nectaries and calycinal glands in *R. ornata* appear to only attract ants on certain parts of the plant. Specifically, in *R. ornata*, ants gather at the petiolar nectaries and calycinal glands and also traverse stems, branches, petioles, and inflorescence axes, but other parts were left ant-free most of the time (i.e., leaf blades and corolla tubes and lobes), leaving herbivores on these areas (tortoise beetles and katydids) undisturbed by ants. In *Ipomoea leptophylla* Torr., the presence of ants on flowers significantly reduced damage from grasshopper herbivores and also decreased seed loss by bruchids [42]. However, ant presence on flowers can also reduce the visitation rates of pollinators, as was reported for *Ipomoea carnea* subsp. *fistulosa* (Mart. ex Choisy) D.F.Austin [116]. This ant–pollinator conflict may be caused directly by aggressive ant behavior deterring pollinators or indirectly by selective plant-trait allocation [117]. While ants were classified as potential protectors, other visitors found on petiolar nectaries and calycinal glands of *R. ornata* (i.e., wasps, cockroaches, and snails) were classified as exudate consumers because they appeared to visit only rarely and did not show any signs of defending the plant. 

Foliar glands were only found on the adaxial lamina of *R. ornata*, but they are present on both leaf surfaces in other species [18,20,22,23,24]. Several studies on foliar glands have suggested a wide range of roles, principally as defense mechanisms, which may also be the case for *R. ornata*. The substances histochemically detected in the foliar glands of *Stictocardia beraviensis* (Vatke) Hallier f. and *S. tiliifolia* (polysaccharides, lipids, terpenes, and flavonoids), are believed to protect plants from herbivores [25]. The mucilaginous exudates found in the foliar glands of *Ipomoea imperati* (Vahl) Griseb. may be responsible for wound healing, provide protection from heat and dehydration, or influence microbial interactions [19]. Moreover, the foliar glands of *Argyreia nervosa* (Burm.f.) Bojer and several species of *Ipomoea* [59,63] were found to have symbiotic relationships with *Periglandula* fungi (Clavicipitaceae). The plants obtained ergot alkaloids from the fungi and stored them in the foliar glands, while providing nutrients and shelter in return [64]. Several studies have suggested that the ergot alkaloids may contribute to chemical defense against herbivores as well as promote plant growth [60,61,62,63,64]. As this mutualism is clade-specific and found in several species that are closely related to *R. ornata* [60,63,118], it offers further research opportunities to determine if the foliar glands of *R. ornata* also influence plant–microbe interactions.

Although the observed petiolar nectaries, calycinal glands, and foliar glands are predicted to provide *R. ornata* with protection from herbivores, we observed several insect species consuming various plant parts in this study. The *Aspidimorpha* tortoise beetles and *Homodes* caterpillars observed consuming leaves may have adaptations against toxic plant metabolites [119,120], particularly since *Aspidimorpha* spp. are known to specialize on Convolvulaceae plant species [121,122]. These herbivores also have adaptations that offer protection against guard ants. *Aspidimorpha* beetles have smooth, shield-like elytra that are difficult for ant mandibles to grasp [123], while caterpillars of *Homodes* sp. are able to avoid guard ants through physical mimicry [124]. Indeed, several species of *Homodes* caterpillars have been reported in close association with weaver ants, mimicking the ants both in terms of morphology and behavior [124,125,126,127,128,129]. Katydids were also observed consuming the corolla lobes that were not guarded by ants. As ants were only observed on the calyx and not the corolla tube and lobes, it is possible that *R. ornata* actually benefits from the absence of ants on corollas. Ants on flowers can deter pollinators and reduce plant reproductive success [116], so the lack of guard ants on exposed floral parts may represent a trade-off, where there is a greater risk of herbivory by florivores during the single night that flowers are open, but they benefit from increased pollinator-visitation rates [116,117,130].

## 5. Conclusions

Our findings provide the first comprehensive details on the external secretory structures of the genus *Rivea* through *R. ornata*, the only species of *Rivea* native to Thailand. The two nectaries function as complex working units (nectary disc formed by nectariferous tissue, epidermis, and vascular tissues; petiolar nectaries formed by nectariferous tissue and epidermis) and also appear to operate in conjunction with non-secreting tissues in the surrounding areas. These two structures, however, secrete nectar in different ways, with the nectary disc releasing nectar through nectarostomata, and the petiolar nectaries employing peltate trichomes. The other secretory structures (i.e., staminal hairs, calycinal glands, and foliar glands) are glandular trichomes. Staminal hairs occur in clumps of long-stalked glands. Calycinal and foliar glands are peltate trichomes and morphologically similar to each other, but the calycinal glands occur in clusters, while the foliar glands are solitarily distributed. Using histochemical methods, all secretory structures were found to produce or accumulate various classes of chemicals (i.e., lipids, phenolic compounds, polysaccharides, terpenoids, flavonoids, and alkaloids); only proteins were absent. Starch grains were only detected in tissues related to the nectary disc and petiolar nectaries, possibly as sources of nectar sugars. Coupling histochemical results with visitor observations led to two hypotheses about their roles: (I) the nectary disc and staminal hairs attract and reward pollinators, and (II) petiolar nectaries, calycinal glands, and foliar glands have roles in plant defense, either against herbivory using plant metabolites and guard ants or against unsuitable environmental conditions (e.g., dehydration and insolation). However, further studies with field experiments are required in order to clarify these hypotheses and assess their contributions to the life history of *R. ornata*.

## Figures and Tables

**Table 1:** Metabolites identified in secretory structures of *Rivea ornata* using histochemical assays.

Metabolite Group	Test	Positive Chromatic Reaction	Nectary Disc		Petiolar Nectaries						
					Glandular Trichome			Epidermis		Nectariferous Parenchyma	
					Head	Stalk	Base				
Total lipids	Sudan black B	Dark blue to black	+, s		+, s	+, s	-	+, s		-	
	Neutral red	Yellow	+		+, s	+, s	-	+, s		-	
General phenolic compounds	Ferric chloride	Brown or black	+, c		+, c	-	-	+, c		+, c	
	Potassium dichromate	Brown	+, c		+, c	-	-	-		-	
Starch	Lugol’s iodine	Dark blue to black	+, c		-	-	-	-		-	
Neutral polysaccharides	Periodic acid–Schiff’s reagent (PAS)	Pink	+, c, s		+, c, s	+, s	+, s	+, c, s		+, c, s	
Acidic polysaccharides	Ruthenium red	Pink to red	+, c, s		+, c, s	+, s	+, s	+, c, s		+, c, s	
Proteins	Mercuric bromophenol blue	Blue	-		-	-	-	-		-	
Terpenoids	Nadi reagent	Dark blue or violet	+, c		+, c	-	-	+, c		+, c	
Flavonoids	Naturstoff reagent A	Yellow	+, c		+, s	-	-	+, s		-	
Alkaloids	Dragendorff reagent	Reddish brown	+, c		+, c	-	-	-		-	
	Wagner’s reagent	Reddish brown	+, c		+, c	-	-	+, c		+, c	
**Metabolite Group**	**Test**	**Positive Chromatic Reaction**	**Calycinal Glands**				**Foliar Glands**			**Staminal Hairs**	
			**Head**	**Stalk**		**Base**	**Head**	**Stalk**	**Base**	**Head**	**Stalk**
Total lipids	Sudan black B	Dark blue to black	+, s	+, s		-	+	+	-	+	+
	Neutral red	Yellow	-	+, s		-	-	+, s	-	+	+
General phenolic compounds	Ferric chloride	Brown or black	+, c	+, c		-	+, c	+	-	-	+, c
	Potassium dichromate	Brown	+, c	+, c		-	+, c	-	-	-	+, c
Starch	Lugol’s iodine	Dark blue to black	-	-		-	-	-	-	-	-
Neutral polysaccharides	Periodic acid–Schiff’s reagent (PAS)	Pink	+	+		+	+, c, s	+, s	+, s	+, s	+, s
Acidic polysaccharides	Ruthenium red	Pink to red	+, c, s	+, c, s		+, s	+, s	+, s	+, s	+, s	+, s
Proteins	Mercuric bromophenol blue	Blue	-	-		-	-	-	-	-	-
Terpenoids	Nadi reagent	Dark blue or violet	+	+		+, c	+	+	-	+, c	+, c
Flavonoids	Naturstoff reagent A	Yellow	+, c, s	+, c, s		-	+	+	-	+, c	+
Alkaloids	Dragendorff reagent	Reddish brown	+, c	+, c		-	+, c	+, s	-	+, c	+
	Wagner’s reagent	Reddish brown	+, c	+, c		-	+, c	-	-	+, c	+, c

Note: -, negative result; +, positive result; c, metabolite clearly detected in cytoplasmic components; s, metabolite clearly detected in structural layers, such as cell walls and/or cell membranes; positive results (+) without c or s labels indicate that the metabolite was detected, but it was unclear whether they were in cytoplasmic components or structural layers.

## Data Availability

Not applicable.
